# An Efficient Markerless Deletion System Suitable for the Industrial Strains of *Streptomyces*

**DOI:** 10.4014/jmb.2106.06083

**Published:** 2021-09-04

**Authors:** Jianxin Dong, Jiaxiu Wei, Han Li, Shiyao Zhao, Wenjun Guan

**Affiliations:** Institute of Pharmaceutical Biotechnology and The Children’s Hospital, Zhejiang University School of Medicine, Hangzhou 310027, P.R. China

**Keywords:** Industrial *Streptomyces*, indigoidine synthetase, large DNA fragment deletion, biosynthetic gene cluster

## Abstract

The genus *Streptomyces* is intensively studied due to its excellent ability to produce secondary metabolites with diverse bioactivities. In particular, adequate precursors of secondary metabolites as well as sophisticated post modification systems make some high-yield industrial strains of *Streptomyces* the promising chassis for the heterologous production of natural products. However, lack of efficient genetic tools for the manipulation of industrial strains, especially the episomal vector independent tools suitable for large DNA fragment deletion, makes it difficult to remold the metabolic pathways and streamline the genomes in these strains. In this respect, we developed an efficient deletion system independent of the episomal vector for large DNA fragment deletion. Based on this system, four large segments of DNA, ranging in length from 10 kb to 200 kb, were knocked out successfully from three industrial *Streptomyces* strains without any marker left. Notably, compared to the classical deletion system used in *Streptomyces*, this deletion system takes about 25% less time in our cases. This work provides a very effective tool for further genetic engineering of the industrial *Streptomyces*.

## Introduction

The Gram-positive *Streptomyces* has been intensively investigated due to their outstanding ability to produce numerous secondary metabolites, many of which are valuable in industrial and pharmaceutical applications [1, 2]. Particularly, some high-yield industrial strains of *Streptomyces* are commonly used for the production of many bioactive natural products (NPs) such as tetracycline (anti-infection), daunorubicin (anti-cancer), and rapamycin (immunosuppressant) [1]. Some of these industrial strains also have the potential to be remolded into suitable cell factories for the heterologous synthesis of valuable pharmaceutical molecules due to abundant precursors supply of NPs as well as sophisticated post modification systems [3].

Most of the industrial strains were, in most cases, poorly characterized physiologically and genetically. This has resulted in a serious limitation of applying molecular genetics approaches since efficient tools have been developed for just a few type strains [4]. High-efficient genetic engineering techniques, such as DNA fragment deletion methods, play a significant role in the research of microbial cell factories. Through DNA fragment deletion, we can explore the functions of essential genes, block the metabolic pathways of by-products, reduce the toxic metabolites, and thereby increase the yield of target products. Unfortunately, most deletion systems are much more difficult to be applied in the industrial strains compared to the best-studied type strains like *Streptomyces coelicolor* M145, *Streptomyces lividans* TK24, and *Streptomyces albidoflavus* J1074 [3, 5], and one important reason for this is the lack of available episomal vectors. In *Streptomyces*, several strategies for mutagenesis have already been developed, specifically, the PCR-targeting system and the CRISPR/Cas9 system, which are two of the most powerful tools. Although CRISPR/Cas9 system enumerated success in several groups for genome editing in *Streptomyces* [6-8], its dependence on available episomal vectors and the inferred toxicity of Cas9 limited its application. For instance, the CRISPR/Cas9 system could not work in several industrial strains including *Streptomyces chattanoogensis* L10, *Streptomyces tsukubaensis* YN06, and *Streptomyces albus* ZD11 [9]. On the other hand, the PCR-targeting system is highly reliable but presents lower efficiency due to the laborious and time-consuming double-crossover mutant screening process [10]. In this context, the chromogenic reporter system for colony screening is a good selection to reduce the time and cost for mutant screening.

Elsewhere, indigoidine synthetase (IDGS) catalyzes the condensation of two L-Glutamine molecules to form one molecule of water-insoluble blue pigment indigoidine [11]. Taking advantage of the blue color of indigoidine, several groups of researchers have realized efficient gene editing based on the IDGS reporter system in *Streptomyces* [10-12]. Li *et al*. used *idgS* gene cloned from *Streptomyces lavendulae* CGMCC 4.1386 to knock out the sco4069 gene in *S. coelicolor* M1146 and the *asfA-y* gene in *Streptomyces* sp. YN86 [11]. Kormanec *et al*. developed an efficient system for markerless deletions and stable integrations in *S. lividans* TK24 based on the *bpsA* gene [10]. Wang *et al*. developed an updated version of the CRISPR/Cas9 genome editing system for actinomycetes based on *IdgS* to address the plasmid curing problem. They performed the deletion of a single gene *actIORFI* in *S. coelicolor* M145 [12]. However, to the best of our knowledge, the applications of the IDGS reporter system primarily in the industrial *Streptomyces* strains remain elusive. Therefore, in this work, an IDGS encoding gene, *sshg_00313* (designated *SaindC* in this study) was cloned from *S. albidoflavus* J1074 [13]. The function of SaIndC was verified, and subsequently, the *SaindC*-based efficient markerless deletion system independent of the episomal vector was constructed. Moreover, three biosynthetic gene clusters (BGCs) and a non-essential chromosomal region were respectively knocked out from three industrial *Streptomyces* strains namely *S. chattanoogensis* L10 [14], *S. coeruleorubidus*, and *S. albus* ZD11[15] based on this deletion system. Collectively, our results indicate that the *SaindC*-based deleting system is efficient and reliable for large DNA fragment deletion in industrial streptomycetes.

## Materials and Methods

### Strains and Vectors

All the strains and vectors used in this study are listed in Table 1.

### Bacterial Cultivation, Fermentation, and HPLC Analysis

All *E. coli* strains were grown at 37°C in Luria-Bertani (LB) medium (1% tryptone, 0.5% yeast extract, and 1%NaCl) [16]. When appropriate, apramycin, kanamycin, or chloramphenicol was added to the media at a ﬁnal concentration of 50, 50, or 25 μg/ml, respectively.

Furthermore, *S. chattanoogensis* L10 and its derivative strains were grown on yeast malt glucose (YMG) agar plates (0.4% yeast extract, 1% malt extract, 0.4% glucose, 0.2% CaCO3, and 2% agar, pH 7.2) for sporulation [17]. 1.75% glucose, 1.5% tryptone, and 1% NaCl was used as the seed broth medium, and YEME broth medium (0.3%yeast extract, 0.3% malt extract, 0.5% tryptone, and 4% glucose) was used for fermentation. In particular, the fermentation process was performed as described previously [14]. Then the production of azoxymycin was analyzed using high-performance liquid chromatography (HPLC) on a Shimadzu LC-20AT system with a UV detector set at 400 nm with Agilent Eclipse Plus-C18 (5μm, 4.6 ×250mm^2^). Mobile phase A was 0.1% formic acid in water, whereas mobile phase B was 0.1% formic acid in acetonitrile. The flow rate was 1 ml min^-1^. During the analysis procedure, mobile phase B was raised from 10 to 90% in 30 min.

Moreover, *S. albus* ZD11, *S. coeruleorubidus*, and their derivative strains were grown on ISP4 agar medium (BD, USA) for 6-7 days at 30°C for sporulation. Tryptic Soy Broth (TSB) medium (3% TSB) was used for pre-cultivation at 30°C for 24 h, industrial seed medium (3% soybean powder, 1% yeast extract, 4% glucose, and 0.2% CaCO3) was used for seed cultivation at 30°C for 29 h, and ionic medium (IM) with soybean oil (0.2% NaCl, 0.2% KCl, 0.5%(NH_4_)_2_SO_4_, 0.02% K_2_HPO_4_, 0.01% MgSO_4_, 0.01% CaCl_2_, 0.5% CaCO_3_, and 15% soybean oil) was used as fermentation medium [18]. The yield of salinomycin was determined as described previously [18]. To determine the production of daunorubicin, the fermentation broth was hydrolyzed by treatment with 39.2 mg of oxalic acid dihydrate per ml at 50°C for 60 min and then mixed with anhydrous methanol at the ratio of 1:9 for 12 h at room temperature. Thereafter, the supernatant was collected using a centrifuge at 12,000 ×*g* for 10 min and then filtered through a 0.22 μm filter. Consequently, the samples were analyzed by HPLC with a ZORBAX Eclipse XDB-C18 column (4.6 × 150 mm^2^, 5 um) on the Shimadzu LC-20AT system, A_254 nm_ was measured. Additionally, mobile phase A was 10 mM ammonium acetate in water, while mobile phase B was acetonitrile. Elution was performed as follows: a linear gradient from 20 to 50% solvent B from 0 to 15 min, a linear gradient from 50 to 90% solvent B from 15 to 18 min, and 90% solvent B from 18 to 22 min, at a flow rate of 1 ml min^-1^.

### Construction of *SaindC*-Based Gene Deletion System

The 3.8 kb *SaindC* gene (Accession: EFE79871.1) was amplified by PCR from *S. albidoflavus* J1074 genomic DNA, while the kasOp* promoter was amplified with primer pair kas-F/kas-R from the plasmid pKC1139S-kasOp previously constructed by our lab. The nucleotide sequence of kasOp* in this plasmid was synthesized by Shanghai Generay Biotech Co., Ltd. (China) according to the previous literature [19]. Afterward, the *SaindC* and kasOp* promoter were inserted into the *Hind*III cleavage site of pSOK804 to generate pINT01. The integrative vector pINT01 containing the kasOp*-*SaindC* cassette and pSOK804 without the cassette was transformed into *E. coli* ET12567/pUZ8002 and conjugated with *S. chattanoogensis* L10 wild-type (WT) strain. After growing on the YMG agar plates containing apramycin for 5 days, the color of the recombinants was recorded. To generate the suicide vector pSUC01, the integrase gene *int* phiC31 was removed from pSET152 by *Hind*III digestion, and then kasOp*-*SaindC* cassette was cloned into the *Hind*III cleavage site by ligation.

### Deletion of Four Large DNA Fragments

The upstream and downstream homologous fragments of the deleting target were amplified with the primers outlined in Table S1. The length of these homologous fragments was 2 kb (for azoxymycin BGC deletion), 2.4 kb (for daunorubicin BGC deletion), 2.2 kb (for salinomycin BGC deletion), and 2.8 kb (for 200 kb non-essential chromosomal region deletion). Consequently, these homologous fragments were inserted into the pSUC01 vector using seamless cloning with the pEASY-Basic Seamless Cloning and Assembly Kit (TransGen Biotech, China) to generate the knockout vector pSUC02, pSUC03, pSUC04 and pSUC05. After confirmed by nucleotide sequencing, the knockout vectors were transformed into *E. coli* ET12567/pUZ8002 and subsequently introduced into *S. chattanoogensis* L10 (pSUC02), *S. coeruleorubidus* (pSUC03), or *S. albus* ZD11 (pSUC04 and pSUC05) through intergeneric conjugation. The recombinants were selected by their resistance to apramycin. Several blue colonies were then transferred to the YMG or ISP4 agar plates without apramycin. Then after growth at 30°C for 4-6 days, the spores were collected and inoculated into the TSB medium. The broth was diluted appropriately and plated on YMG or ISP4 plates to form the single colonies after 2 days. Within 4-6 days of cultivation, the numbers of blue and original color colonies were recorded. To analyze the genotype of the recombinants, the original color colonies on the plates were transferred to a new plate for further growth at 30°C. Lastly, 4-6 days later, the mycelia were inoculated into TSB medium and grown at 30°C for 24 h, the genomic DNA was then extracted and used as PCR template.

### Bioinformatics Analysis

To determine the non-essential regions on *S. albus* ZD11, whole genome alignment of the genome of *S. albus* ZD11 with other six *Streptomyces* strains were performed with MUMmer [20], chromosomal sequences of *S. albidoflavus* J1074 (CP004370.1), *S. coliecolor* A(3)2 (NC_003888.3), *S. lividans* TK24 (NZ_CP009124.1), *S. clavuligerus* ATCC 27064 (NZ_CP027858), *S. griseus* NBRC 13350 (NC_010572), and *S. avermitilis* MA-4680 (NC_003155) were obtained from NCBI. Moerover, in order to determine the BGCs, genomic islands (GIs), integrative and conjugative elements (ICEs), and inserted sequences (ISs), computational analysis were performed using antiSMASH [21], Island viewer4 [22], and ISsaga2 [23]. The detail of the transcriptome sequencing (RNA-seq) data of WT *S.albus* ZD11 mentioned in the results part was described in our previous work [18], the sequence reads obtained by RNA-seq were deposited in the SRA database under accession numbers SAMN12251778, SAMN12251779.

## Results

### Construction of *SaindC*-Based Markerless Gene Deletion System

**Fig. 1:**
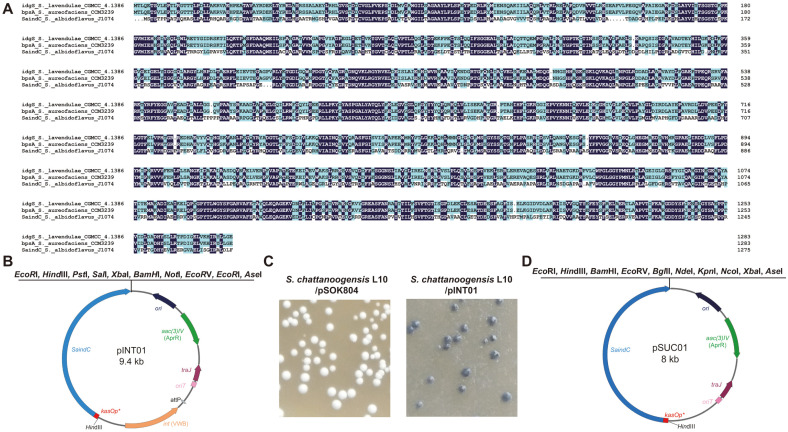
Construction of the *SaindC*-based markerless deletion system. (**A**) The amino acid sequence identity among *idgS* from *S. lavendulae* CGMCC 4.1386, *bpsA* from *S. aureofaciens* CCM3239, and *SaindC* from *S. albidoflavus* J1074. (**B**) Organization of the integrative vector pINT01. (**C**) The recombinants carrying pSOK804 produced no indigoidine, while the recombinants carrying pINT01 produced indigoidine on the YMG agar plates. (**D**) Organization of the suicide vector pSUC01.

Although the indigoidine BGC was noted to be activated in *S. albidoflavus* J1074 [13], the indigoidine synthetase gene (*SaindC*) located in this BGC has never been used in any reporter system. The *SaindC* presents 87.73% amino acid sequence identity to *idgS* or *bpsA* which had been used as a reporter gene in *Streptomyces* (Fig. 1A) [10, 11, 24, 25]. To verify the function of SaIndC, *SaindC* from *S. albidoflavus* J1074 was cloned into an integrative vector pSOK804 under control of the kasOp* promoter [19, 26]. The resulting vector pINT01 (Fig. 1B) and pSOK804 were then introduced into *S. chattanoogensis* L10 by conjugation. The recombinants were cultured on YMG agar plates for 5 days, where the blue indigoidine was observed in the recombinants of L10/pINT01, but not in the recombinants of L10/pSOK804 (Fig. 1C). This implies that the kasOp*-*SaindC* cassette can be used as a reporter system.

To achieve markerless large-fragment deletion in *Streptomyces*, the suicide vector pSUC01 derived from pSET152 was constructed [27], in which the integrase gene *int* phiC31 was deleted while the kasOp*-*SaindC* cassette was inserted. For the convenience of the cloning of homologous fragments, the multi-cloning sites were redesigned near the kasOp*-*SaindC* cassette (Fig. 1D).

**Fig. 2:**
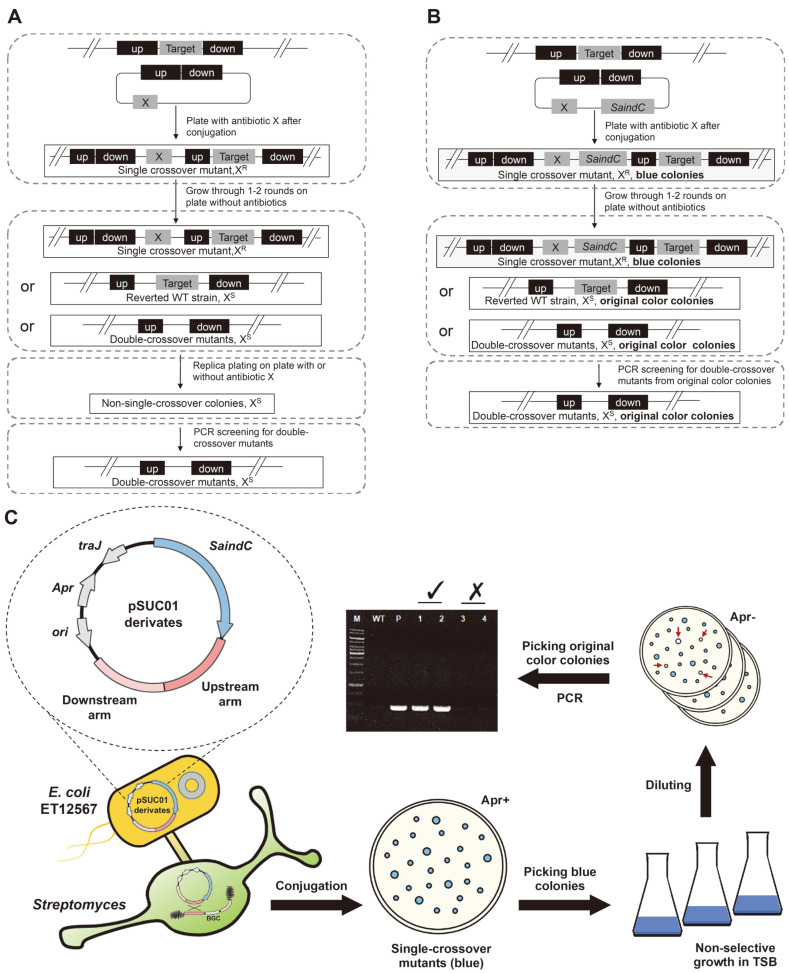
Schematic diagram of the DNA fragment deletion process. Comparison of the the classical (**A**) and the *SaindC*-based (**B**) deletion process in *Streptomyces*. X, resistance gene cassette for antibiotic X; up, the upstream homologous arm of the target DNA fragment; and down, the downstream homologous arm of the target DNA fragment. (**C**) The flow of *SaindC*-based deletion process. Apr+, plates with apramycin added; Apr-, plates without apramycin added; original color colonies are indicated with red arrows.

For the traditional markerless deletion system in *Streptomyces*, a time-consuming replica plating step is required to distinguish the single-crossover mutants that are resistant to the specific antibiotic from the sensitive non-single-crossover colonies (reverted wild-type or double-crossover mutants, Fig. 2A). In our method, with the *SaindC*-based reporter system, the colonies of single-crossover mutant (blue), double-crossover mutant (original color), and reverted wild-type (original color) can be easily characterized based on the colony color with no need for replica plating (Figs. 2B-2C).

### Deletion of the 10 kb Azoxymycin BGC in *S. chattanoogensis* L10

**Fig. 3:**
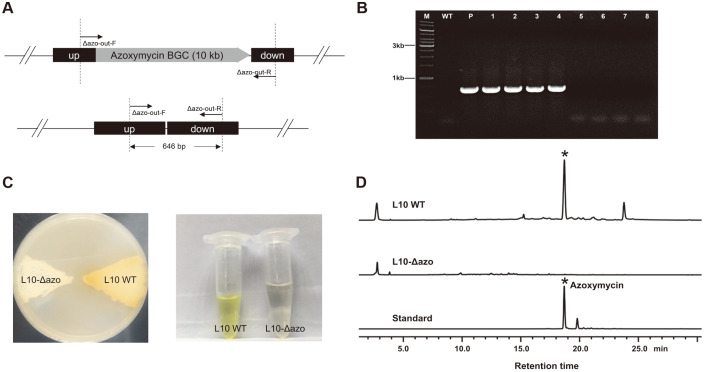
Deletion of the azoxymycin BGC in *S. chattanoogensis* L10. (**A**) The schematic diagram of the doublecrossover mutants. Up, the upstream homologous arm of azoxymycin BGC; while down, the downstream homologous arm of azoxymycin BGC. The primer pair Δazo-out-F/Δazo-out-R was used for PCR verification. (**B**) PCR verification of the L10- Δazo mutants. Lane M, DNA marker; lane WT, wild-type *S. chattanoogensis* L10; lane P, pSUC02; lane 1-4, double-crossover mutants (4 colonies that secreted no yellow azoxymycin into the plate); lane 5-8, reverted wild-type colonies (4 randomly selected colonies that secreted yellow azoxymycin into the plate). (**C**) Color comparisons of substrate mycelia and fermentation broth extract between the L10-Δazo mutant and L10 WT strain. The strains were cultured for 5 days on the YMG agar plate before being photographed. (**D**) The HPLC analysis of the fermentation broths at 120 h.

*S. chattanoogensis* L10 is an industrial natamycin-producing strain [14], whereas the yellow azoxymycin is a byproduct in the natamycin production process. The 10 kb azoxymycin BGC had been cloned and characterized in 2015 [28]. Based on a proof-of-concept of the feasibility and efficiency of the *SaindC*-based markerless deletion system, this relatively small BGC was selected as our first target to delete (Fig. 3A).

The suicide vector pSUC02 was used for deleting this BGC. After conjugation, only blue colonies (single-crossover mutant) were noted on the plates containing apramycin, and thus no PCR validations were required. Three randomly selected blue colonies were collected and streaked onto the YMG agar plates without apramycin to ensure the occurrence of the second homologous recombination (HR). Following two rounds of non-selective growth, 644 colonies were identified from 22 plates, and 94 out of 644 (15%) colonies were white. This signifies that 85% of the colonies were still single-crossover mutants (blue) and could be excluded simply based on the color of the colony, and hence 4-5 days for the replica plating step was saved. In addition, among the 94 white colonies, 39 colonies produced yellow substance (wild-type revertant) and the other 55 colonies did not (double-crossover mutants), four white colonies without yellow substance secreted into the plate and four white colonies that generated yellow substance into the plate were randomly selected and verified using PCR with primer pair Δazo-out-F/Δazo-out-R. The PCR finding revealed that all of the four colonies that secreted no yellow substance were double-crossover mutants (Fig. 3B).

Furthermore, one randomly selected double-crossover mutant (designated L10-Δazo) was cultured on a YMG plate, while the cultivation and fermentation procedure was performed as described above. As shown in Fig. 3C, L10-Δazo produced no yellow substance on the plate or in the fermentation broth. Notably, the HPLC analysis result elucidated that no azoxymycin was detected in the L10-Δazo mutant (Fig. 3D), indicating that the 10 kb azoxymycin BGC has been successfully deleted.

### Deletion of the 37 kb Daunorubicin BGC in *S. coeruleorubidus*

**Fig. 4:**
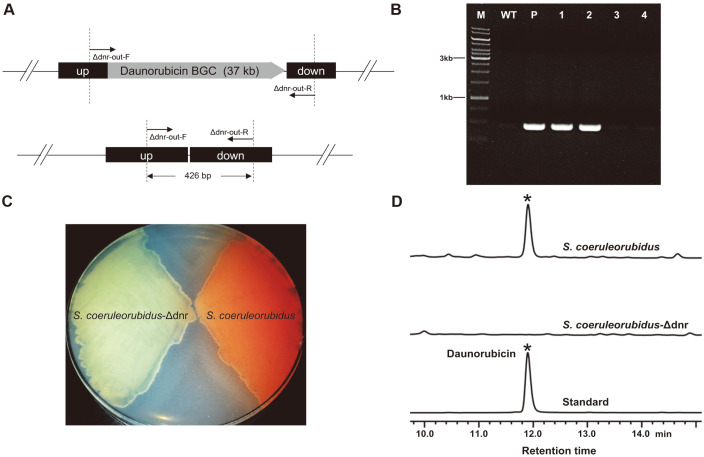
Deletion of the daunorubicin BGC in *S. coeruleorubidus*. (**A**) The schematic diagram of the double-crossover mutants. Up, the upstream homologous arm of the daunorubicin BGC; down, the downstream homologous arm of the daunorubicin BGC. The primer pair Δdnr-out-F/Δdnr-out-R was used for PCR verification. (**B**) PCR verification of the *S. coeruleorubidus*-Δdnr mutants. Lane M, DNA marker; lane WT, wild-type *S. coeruleorubidus*; lane P, pSUC03; lane 1-2, doublecrossover mutants (2 randomly selected colonies that secreted no light red pigments into the plate); lane 3-4, reverted wild-type colonies (2 randomly selected colonies that produced the light red compounds into the plate). (**C**) Color comparison of the substrate mycelium cultured for 6 days before being photographed. (**D**) The HPLC analysis of the fermentation broths at 120 h.

The *S. coeruleorubidus* strain used in this study is an industrial daunorubicin-producing strain purchased from China Center of Industrial Culture Collection (CICC). To examine the universal applicability of this markerless deletion system and test its ability to deleting larger gene clusters, a 37 kb daunorubicin BGC in *S. coeruleorubidus* was chosed as our next target (Fig. 4A). After conjugation and non-selective growth, it was observed that the proportion of blue colonies (single-crossover mutants) was 47.74% among all 15 plates, the rest 52.26% colonies exhibited the original color of *S. coeruleorubidus*. Eight random selected colonies with original color were transferred to another agar plate for sporulation. Five days later, it was found that 6 colonies secreted light red pigment (the color of daunorubicin) into the plate while the other 2 colonies yielded no color change. The PCR result suggested that those two colonies were daunorubicin BGC knockout mutants (Fig. 4B). Subsequently, the verified daunorubicin BGC knockout mutants and wild-type *S. coeruleorubidus* were grown on the ISP4 plate (Fig. 4C), and then fermentation procedures and chromatographic analysis assay were executed. The HPLC results further confirmed the deletion of the daunorubicin BGC, no daunorubicin was detected in the knockout mutants (Fig. 4D).

### Deletion of the 74 kb Salinomycin BGC in *S. albus* ZD11

*S. albus* ZD11 is a derivative of an industrial high-yielding salinomycin-producing strain [15]. The high yield of salinomycin suggests that *S. albus* ZD11 can provide abundant acyl-CoA precursors for polyketide synthesis and has good potential to be an efficient microbial chassis for PKS synthesis. However, due to the lack of available episomal expression vectors, the CRISPR/Cas9 system could not be used in this strain. To further examine the universal applicability of this markerless deletion system, the 74 kb salinomycin BGC was selected as our third target to delete.

**Fig. 5:**
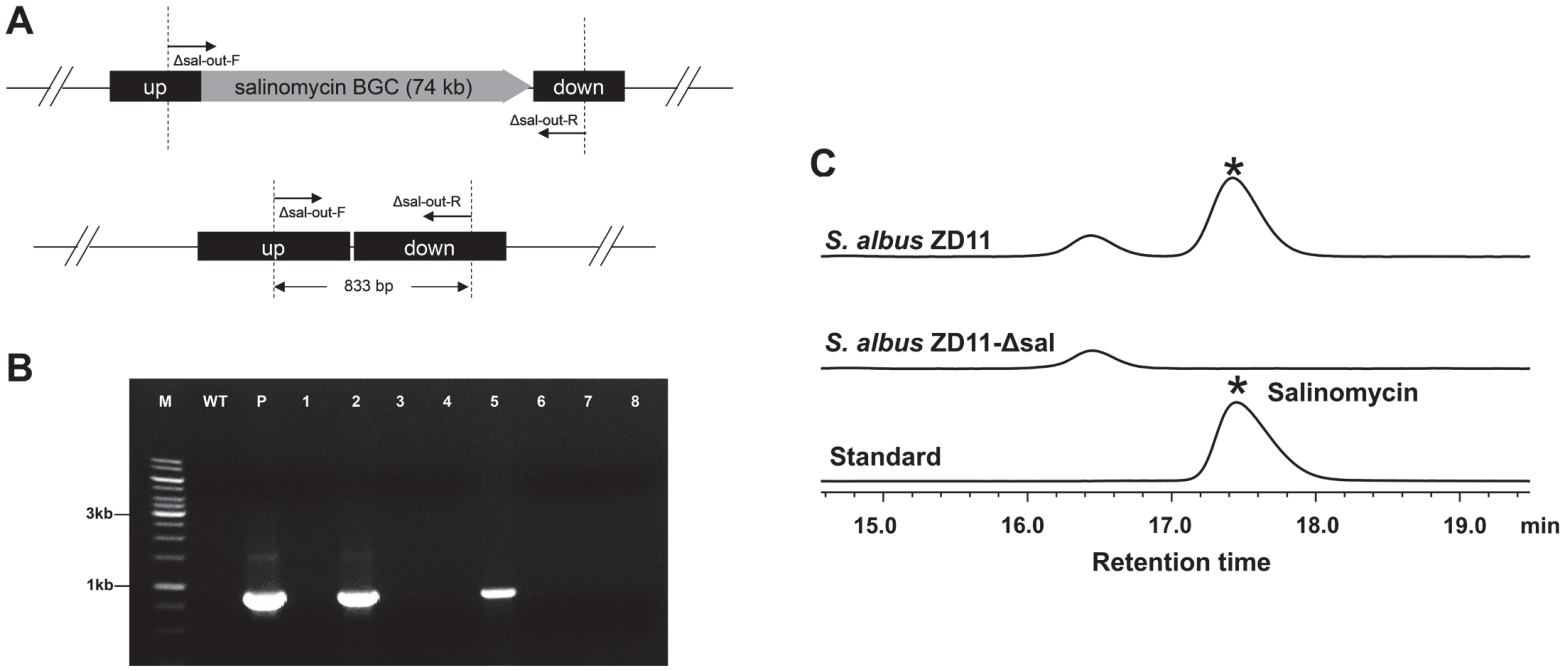
Deletion of the salinomycin BGC in *S. albus* ZD11. (**A**) The schematic diagram of the double-crossover mutants. Up, the upstream fragment of the target BGC; down, the downstream fragment of the target BGC. The primer pair Δsal-out-F/Δsal-out-R was used for PCR verification. (**B**) PCR verification of *S. albus* ZD11-Δsal. Lane M, DNA marker; lane WT, wild-type *S. albus* ZD11; lane P, pSUC04; lane 2 and lane 5, double-crossover mutants; lane 1, 3, 4, 6, 7, 8, reverted wild-type colonies. (**C**) The salinomycin production was detected using HPLC at 120 h.

Similarly, two 2.2 kb homologous fragments flanking salinomycin BGC were inserted into pSUC01 to generate pSUC04. Then the vector was introduced into *S. albus* ZD11 through conjugation to delete the target large fragment (Fig. 5A). After non-selective growth, 32.66% of the colonies among 10 plates appeared to be blue, the rest 67.34% colonies exhibited to be white (the original color of *S. albus* ZD11). Eight white colonies were randomly selected and verified using PCR with primer pair Δsal-out-F/Δsal-out-R, if the whole gene cluster was deleted, an 833 bp PCR product would be obtained. The findings revealed that 2 out of 8 white colonies were double-crossover mutants (Fig. 5B), which were designated as ZD11-Δsal-1 and ZD11-Δsal-2. In addition, the ZD11-Δsal-1 mutant and the *S. albus* ZD11 WT strain were cultured in the fermentation medium, respectively. The HPLC results demonstrated that salinomycin was not detected in the fermentation broth of the ZD11-Δsal-1 mutant (Fig. 5C), which further implies the successful deletion of salinomycin BGC.

### Deletion of 200 kb Non-Essential Chromosomal Region in *S. albus* ZD11

**Fig. 6:**
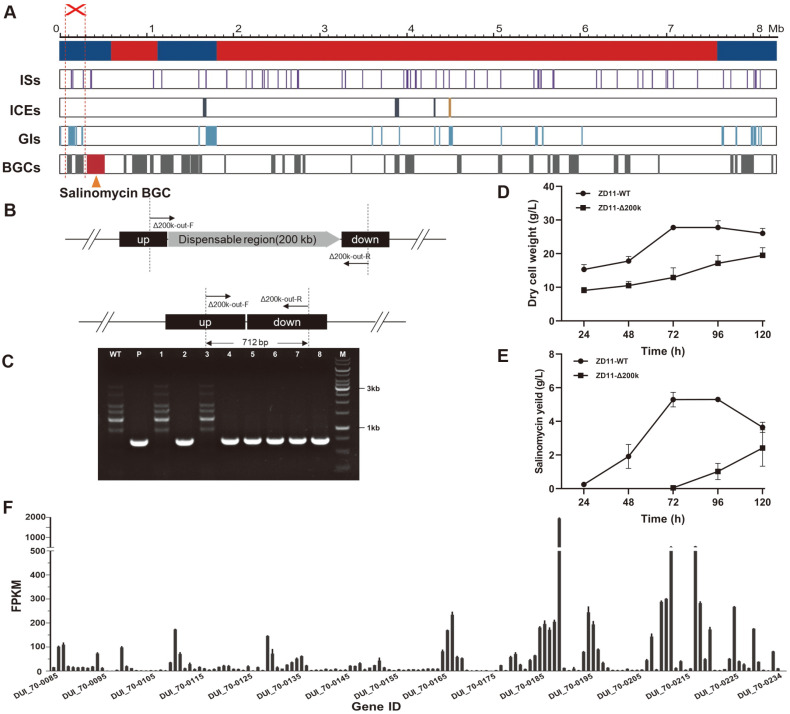
Deletion of the 200 kb chromosomal region in *S. albus* ZD11. (**A**) Mapping of essential (red color) and nonessential (blue color) regions and localization of ISs, ICEs, GIs, and BGCs in the genome of *S. albus* ZD11. (**B**) The schematic diagram of the double-crossover mutants. Up, the upstream fragment of the target BGC; down, the downstream fragment of the target BGC. The primer pair Δsal-out-F/Δsal-out-R was used for PCR verification. (**C**) PCR verification of *S. albus* ZD11- Δsal. Lane M, DNA marker; lane WT, the wild-type *S. albus* ZD11; lane P, pSUC05; lane 1 and lane 3, the reverted wild-type strains; lane 2, 4, 5, 6, 7, 8, the double-crossover mutants. The growth curves (**D**) and salinomycin production curve (**E**) of mutant and *S. albus* ZD11 WT strain. (**F**) The expression level of the genes in the 200 kb deleted region.

Large-scale genome reduction can not only decrease metabolic burden on host cell, but can also further develop simplified and versatile chassis for NPs production. Therefore, development of efficient large fragments deletion methods is of great importance. In this part, the essential regions (600,000-1,139,003 bp, and 1,827,705-7,635,294 bp) and non-essential regions (1-600,000 bp, 1,139,004-1,827,704 bp, and 7,635,295-8,317,371 bp) were predicted through whole-genome alignment (Fig. 6A), and a 200 kb non-essential chromosomal region of *S. albus* ZD11 (91,690-291,933 bp), which containg 150 predicted open reading frames, two BGCs, five ISs and six GIs, were selected as the deleting target (Fig. 6A), to further examine the applicability of this markerless deletion system.

After the conjugation and non-selective growth, 92% of the colonies among six plates appeared to be blue (single-crossover mutants), the rest 8% colonies exhibited to be white (the original color of *S. albus* ZD11). Eight white colonies were randomly seletcted and verified by PCR, and six of them were confirmed as the double-crossover mutants (Figs. 6B-6C). It was shown that deletion of the 200 kb region resulted in significant reduction of cell growth and salinomycin production (Figs. 6D-6E). According to the RNA-seq data of WT *S.albus* ZD11, although the overwhelming majority of genes located in this region were low expressed (Fig. 6F), 22 genes exhibited exceptionally higher expression levels (FPKM >100). The function of seven of them were unknown and the others were mainly responsible for secondary metabolism. Specifically, the expression level of DUI_70-0189, which encoding ABC-type Fe^3+^-siderophore transporter substrate-binding protein, was particularly high (The FPKM value is 1955). Previous studies suggested that iron are required for the vast majority of bacteria to survive, and siderophore is vital for bacteria to uptake iron from the environment [29]. Based on this, we speculate that the deletion of DUI_70-0189 might be one of the causes for the reduction of cell growth and salinomycin production.

## Discussion

In this paper, we explored the possibility of using *SaindC*, an indigoidine synthetase gene from *S. albidoflavus* J1074, to construct an efficient *SaindC*-based markerless deletion system which has good application prospect in the deletion of BGCs and other large DNA fragments from industrial *Streptomyces*.

In the process of genomic fragment deletion through double-crossover HR, the second HR occurred in the non-selective growth step and may generate double-crossover mutants or reverted wild-type. For the classical selection methods without a reporter, the laborious and time-consuming replica plating and PCR verification are required to screen the double-crossover mutants. However, a large part of the colonies on the plates after non-selective growth are single-crossover mutants, which significantly decrease the ratio of the correct double-crossover mutants. With our *SaindC*-based reporter system, the colonies of single-crossover mutant (blue), double-crossover mutant (original color), and reverted wild-type (original color) can be easily characterized based on the color of colonies (Fig. 2C). Furthermore, by skipping the laborious replica plating step, the total time needed for deletion was averagely reduced with about 25% in our cases (5-7 days were reduced for different strains).

Using the indigoidine synthetase genes, several groups have previously reported efficient gene-deletion in the model *Streptomyces* strains including *S. coelicolor* M145, *S. coelicolor* M1146, and *S. lividans* TK24 [10-12]. However, the feasibility of their deletion systems was not evaluated in industrial strains in their works. Here, the indigoidine-based markerless deletion system was tested in three industrial strains, and the results elucidated that our method provide a choice for many industrial strains particularly those lacking the episomal vectors.

The most notable characteristic of *Streptomyces* is its ability to produce secondary metabolites. Nevertheless, many BGCs are silent under current culture conditions, thus heterologous expression has become a major strategy to obtain enough such metabolites. In particular, industrial streptomycetes have been proposed as suitable heterologous expression hosts because of adequate precursors as well as sophisticated post modification systems [3]. To improve the yield of heterologous products, the non-essential chromosomal regions like endogenous secondary BGCs, GIs and ISs are usually deleted ahead, there is an urgent requirement for reliable and efficient large DNA fragment deletion systems. Herein, the 10 kb, 37 kb, 74 kb, and 200 kb DNA fragments were deleted efficiently with our system, thus implying that this system is at least not constrained by the size of the target genomic fragment in the range of 10-200 kb. As the indigoidine synthetase could also be used in most actinomycetes as well as in some rare actinomycete genus [10, 11, 25], our work may provide a versatile markerless deletion tool for several industrial actinomycete strains.

## Supplemental Materials

Supplementary data for this paper are available on-line only at http://jmb.or.kr.

## Figures and Tables

**Table 1:** Bacterial strains and vectors used in this work.

Strains or vectors	Description	Reference
Strains		
*S. albidoflavus* J1074	Type strain of *Streptomyces*	[13]
*S. chattanoogensis* L10	Industrial natamycin producing strain	CGMCC 2644
*S. chattanoogensis* L10/ pINT01	L10 carrying vector pINT01, apr	This study
*S. chattanoogensis* L10/ pSOK804	L10 carrying vector pSOK804, apr	This study
*S. chattanoogensis* L10-Δazo	L10 with disruption of azoxymycin BGC	This study
*S. albus* ZD11	A derivative obtained with streak plate method from an industrial salinomycin-producing strain	CGMCC 4.7658
*S. albus* ZD11-Δsal	ZD11 with disruption of salinomycin BGC	This study
*S. albus* ZD11-Δ200k	ZD11 with disruption of 200 kb non-essential chromosomal region deleted	This study
*S. coeruleorubidus*	Daunorubicin producing strain purchased from CICC	CICC 11043
*S. coeruleorubidus* -Δdnr	*Streptomyces coeruleorubidus* with disruption of daunorubicin BGC	This study
*E.coli* TG1	Host strain for DNA clone	Stratagene
*E.coli* ET12567 (pUZ8002)	dam^-^dcm^-^ strain containing helper plasmid pUZ8002	[30]
Vectors		
pKC1139S-kasOp	Dereived from pKC1139 containg kasOp*, apr	Unpublished
pSOK804	*Streptomyces* / *E. coli* shuttle vector, apr	[31]
pINT01	Derived from pSOK804 containing kasOp*-*SaindC* cassette, apr	This study
pSET152	*Streptomyces* / *E. coli* shuttle vector, apr	[27]
pSUC01	Derived from pSET152 containing kasOp*-*SaindC* cassette, apr	This study
pSUC02	Derived from pSUC01 for the deletion of azoxymycin BGC, apr	This study
pSUC03	Derived from pSUC01 for the deletion of daunorubicin BGC, apr	This study
pSUC04	Derived from pSUC01 for the deletion of salinomycin BGC, apr	This study
pSUC05	Derived from pSUC01 for the deletion of the 200 kb non-essential chromosomal region, apr	This study

apr, apramycin resistance
