# Supplementary material for: An Efficient Markerless Deletion System Suitable for the Industrial Strains of Streptomyces
Source: J Microbiol Biotechnol. 2021 Sep 4;31(12):1722–31. doi: 10.4014/jmb.2106.06083 (PMC9705919; doi:10.4014/jmb.2106.06083)
Supplement: Supplementary file 1 [file jmb-31-12-1722-supple.pdf]

J. Microbiol. Biotechnol.
https://doi.org/10.4014/jmb.2106.06083

An efficient markerless deletion system suitable for the industrial strains of *Streptomyces*

Jianxin Dong1, Jiaxiu Wei1, Han Li1, Shiyao Zhao1, Wenjun Guan1*

1 Institute of Pharmaceutical Biotechnology and The Children's Hospital, Zhejiang University School of Medicine, Hangzhou, China

*Corresponding author: guanwj@zju.edu.cn

Tel: +86-0571-88206477

Supplementary materials

Table S1 Primers used in this study

Name	Sequence (5'-3')
SaindC-F	ATGAGCACCAGCACGCCGCC
SaindC-R	CTAGGCGAAGAGGTCCAAGG
SaindC-F-2	GTGCAGGACTGGGGGAGTTATGAGCACCAGCACGCCGCC
SaindC-R-2	aagctTGAATTcCTAGGCGAAGAGGTCCAAGG
kas-F	gagggcgcgaagcttTGTTCACATTCGAACGGTCT
kas-R	CGTGCAGGACTGGGGGAGTT
azo-up-F	gacctcttcgcctaggaattcGCGCCTGTCTGAGCGTAACT
azo-up-R	tttcccagaaggcctgtacgAGTGCCGGCCAGACCAACTA
azo-down-F	CTGGCCGGCACTCGTACAGGCCTTCTGGGAAA
azo-down-R	agaccatggggtacccatatgGGCCTTGCGATGGATATCCC
Δazo-out-F	CGGAGGTGAGGCGTCAGTGT
Δazo-out-R	TCCTCCCACCAGAGCAGGCT
dnr-up-F	taggaattcaagcttggatccAGATCCTGCTCACCCGTGTCT
dnr-up-R	tcatcgtcatACCATCCGAGTCGTGATTGCT
dnr-down-F	ctcggatggtATGACGATGACCGAGTTCCCCTT
dnr-down-R	agaccatggggtacccatatgCGCTGAGCAAACCCCAGTAGCC
Δdnr-out-F	CCCTCCCGCTCGTGCCTGT
Δdnr-out-R	AGGTTGCCAAGACCCTCCGTTC
sal-up-F	agaccatggggtacccatatgGCTCCCGCTGGAACGGGTAG
sal-up-R	acatggacggcatgctcgacGTTTAAACCCACGAGGACCCGGTCGTCA
sal-down-F	GTCGAGCATGCCGTCCATGT
sal-down-R	ttcaagcttggatccgatatcGGGCTGCGCGGACACCTGAC
Δsal-out-F	AGCACCTCGTGGCCCAACTCG
Δsal-out-R	CTGCGCGTCAGCCATGCCTT

Δ200k-out-F	CGCCGGGTGAACGAGGACAG
Δ200k-out-R	ACGTGGTGGAGCAGGGCTAC

Up-F and up-R are primers for constructing the upstream fragment of the target gene, and down-F and down-R for constructing the downstream fragment. Out-F and out-R were used for the verification of the deletion of the target genes

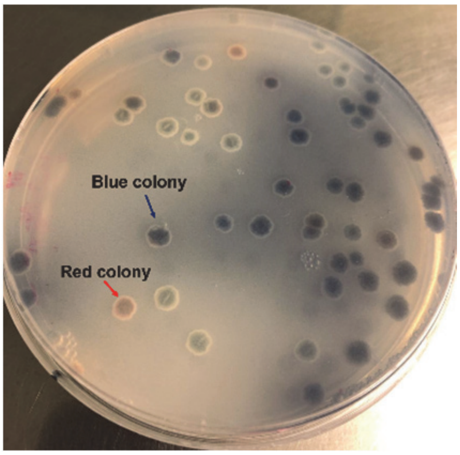

Figure S1. The indigoidine production of doxorubicin-producing *S. coeruleorubidus* strain. The red colonies are the wild-type *S. coeruleorubidus* without indigoidine produced, the blue colonies are the mutants producing indigoidine.
